# Case review of 3 patients with musculoskeletal manifestation of human immunodeficiency virus and highly active antiretroviral therapy in the form of osteopenia and insufficiency fractures

**DOI:** 10.1093/bjrcr/uaaf044

**Published:** 2025-09-09

**Authors:** Jainam A Doshi, Pushpa Bhari Thippeswamy

**Affiliations:** Department of Radiology, Ganga Hospital, Coimbatore, 641043, India; Department of Radiology, Ganga Hospital, Coimbatore, 641043, India

**Keywords:** HAART, tenofovir-associated osteopenia, osteopenia, insufficiency fractures, vertebral osteonecrosis, osteopenia related to HIV

## Abstract

Patients infected with human immunodeficiency virus are prone to develop multiple complications related to the musculoskeletal system with aetiologies including infection, inflammatory, neoplastic, and coagulopathy, of which infection is the most common.^1^ The underlying mechanisms leading to these diseases are complex and multifactorial. Antiretroviral drugs have helped to reduce the burden of complications; however, they may pose several adverse effects. We have tried to draw attention to the impact of intake of nucleoside reverse transcriptase inhibitor-class drugs (like tenofovir), which is implicated in severe osteoporosis, multiple insufficiency fractures, and osteonecrosis.^2^^,^^3^ Radiology plays an important role in early diagnosis and treatment planning in these patients, in whom clinical and laboratory findings are commonly equivocal and non-specific.

## Introduction

Patients infected with human immunodeficiency virus are prone to develop multiple complications related to the musculoskeletal system with aetiologies including infection, inflammatory, neoplastic, and coagulopathy, of which infection is the most common.^1^ There is an increasing number of cases of human immunodeficiency virus (HIV) worldwide. As per World Health Organization, there will be 39.9 million PLWH (people living with HIV) by the end of 2023, of which 1.3 million were new infections in 2023. Increasing awareness and education about the disease has resulted in early diagnosis and usage of highly active antiretroviral therapy (HAART) to reduce the disease burden and improve life expectancy. HIV infection itself is known for its immune modulation resulting in a flare of cytokines in the plasma causing multiple ill effects in the body, decreased bone mineral density (BMD) is among the common ones. Certain HAART drugs are implicated in further reducing BMD. We are presenting 3 case scenarios highlighting the implications of HAART on bone density and related complications.

## Case 1

A 58-year-old gentleman presented to orthopaedic Out patient department (OPD) with chief complaints of bilateral ankle swelling and pain for 6 months. The patient was a known case of HIV infection for 15 years. He was a non-smoker and non-alcoholic with no other comorbidities like hepatitis B or C infection or hyperparathyroidism.

The physical examination revealed swelling around the ankle joint and pain throughout the full range of motion. A clinical suspicion of infectious or inflammatory arthropathy was raised. The osteoporosis profile showed a mild vitamin D deficiency. The inflammatory and infective profiles, such as serum uric acid, RA factor, C-reactive protein (CRP), anti-CCP antibody, and Erythrocyte sedimentation rate (ESR) were normal.

**Figure 1.**
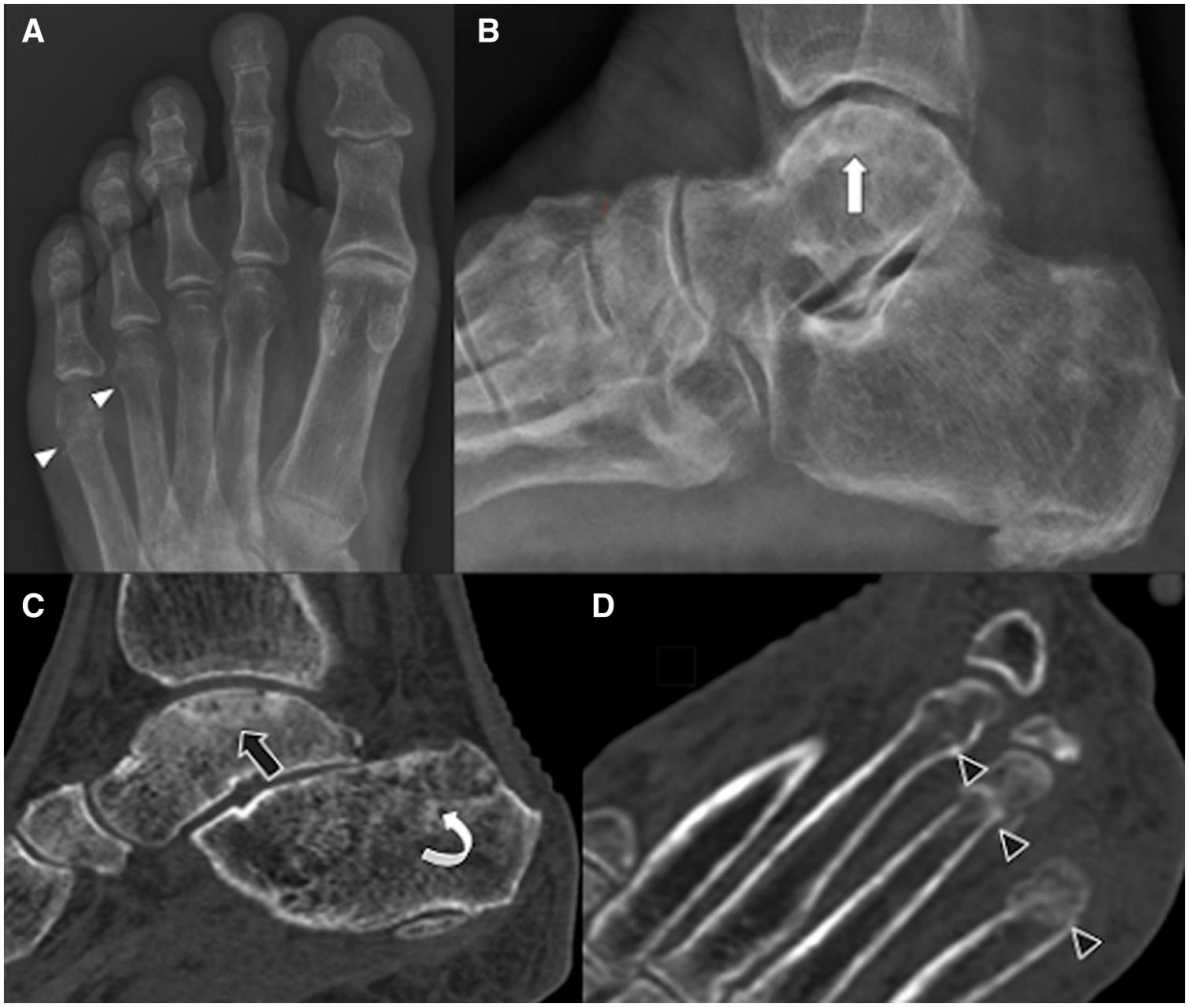
Plain radiographs and CT scan images show generalized reduction in bone density and stress fractures**—**generalized reduction in bone density and fracture of fourth and fifth metatarsals distal shaft (white arrowheads) on AP view radiograph (A), sclerosis with irregularity of the articular surface of the talus dome with maintained tibio-talar joint space on lateral view radiograph (B), reduced bone mineral density, subchondral fracture of the talus dome with surrounding sclerosis (black arrows) and fracture of the calcaneus (curved arrow) on sagittal plane CT scan (C), fracture of distal shaft of third, fourth, and fifth metatarsals (black arrowheads) on axial plane CT scan (D).

**Figure 2.**
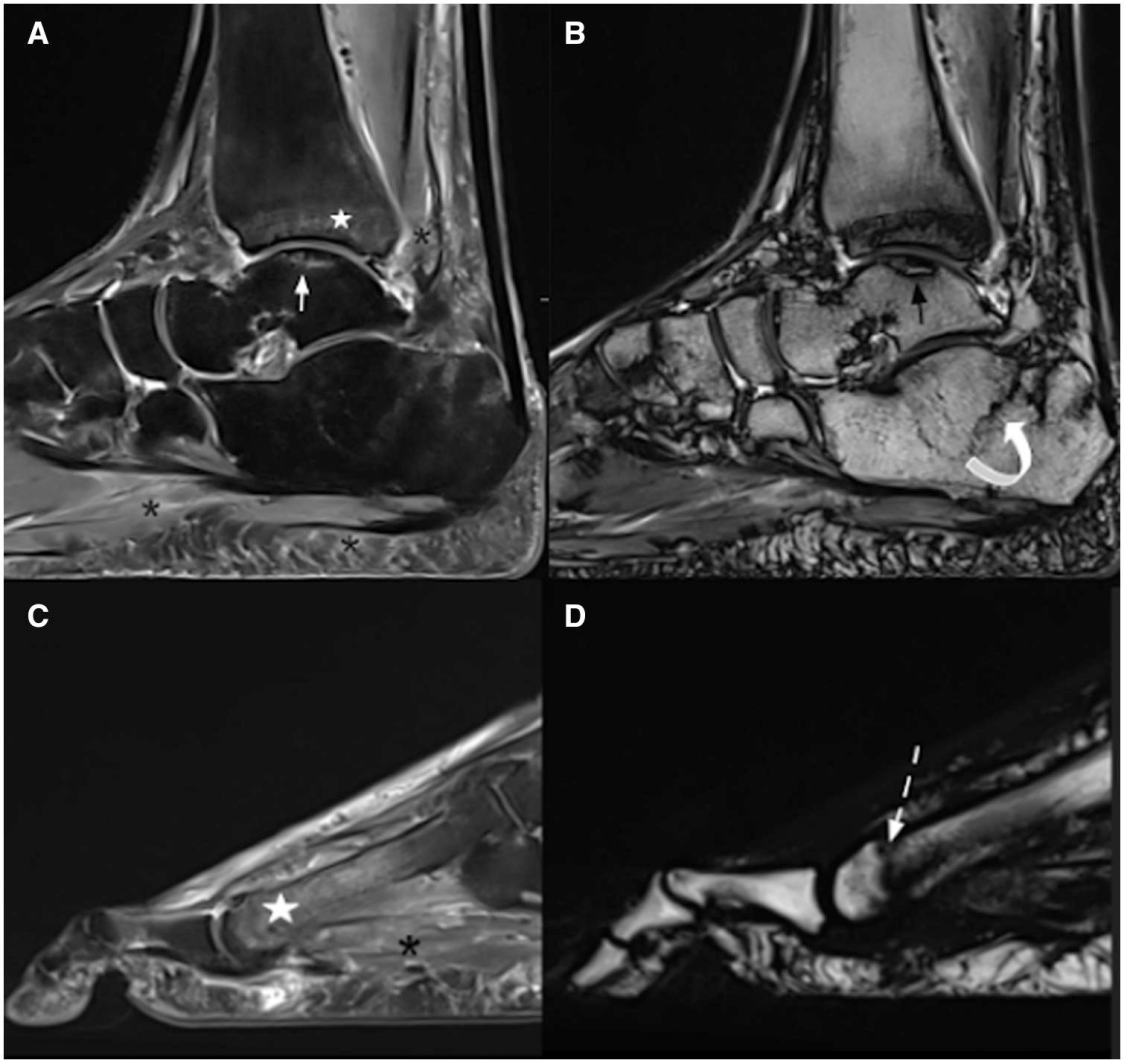
MRI images of the left ankle and foot shows talar dome and metatarsal distal shaft fractures soft tissue and bone marrow oedema—fracture of the talar dome (white arrow), marrow oedema in distal tibia (star), and soft tissue and kager’s fat pad oedema (asterisk) on sagittal PDFS image (A), subchondral insufficiency fracture of the talar dome with surrounding sclerosis (black arrow) and hypointense fracture line in calcaneus (curved arrow) on sagittal T2W image (B), oedematous changes in the shaft of metatarsal (star) and oedematous changes in surrounding soft tissue (asterisk) on sagittal PDFS image (C), hypointense fracture line in distal shaft of metatarsal (dashed arrow) on sagittal Dixon fat only image (D).

*Imaging*: Plain radiograph showed reduced BMD, sclerosis with articular surface irregularity of the talus dome with maintained tibio-talar joint space, and fracture of distal shaft of the fourth and fifth metatarsals (Figure 1). However, due to significant soft tissue swelling clinically, MRI was performed to rule out infective or inflammatory process. MRI revealed a subchondral fracture of the medial talar dome with mild depression and a fracture line with intact overlying cartilage. There were also linear fractures of the distal shaft of third, fourth, and fifth metatarsal bones with diffuse marrow oedema in the rest of the metatarsal shaft and moderate soft tissue oedema around the fractured bones. Un-displaced fracture line is also seen in the calcaneus. Marrow oedema was seen in the subchondral region of the tibia. Diffuse subcutaneous soft tissue oedema and Kager’s fat pad oedema indicated reactionary changes (Figure 2). On CT correlation, there was generalized reduction in the bone density and fractures as seen on MRI (Figure 1).

After analysing the history, clinical examination, laboratory investigations, and imaging features, osteoporosis with insufficiency fractures was proposed as a final diagnosis.

## Case 2

A 47-year-old lady presented to orthopaedic OPD with chief complaints of left lower limb pain for 4 months. The pain was insidious in onset, gradually progressive, and associated with left lower limb radiculopathy. No history of weakness or numbness in the lower limbs or bladder/bowel disturbances. No history of trauma/fever/weight loss/loss of appetite. The patient was a known case of HIV infection for 18 years. She was a non-smoker and non-alcoholic with no other comorbidities like hepatitis B or C infection or hyperparathyroidism. Physical examination showed tenderness over the lower lumbar region on palpation. A clinical suspicion of infective spondylodiscitis was raised. The osteoporotic profile, infective, and inflammatory markers were normal.

**Figure 3.**
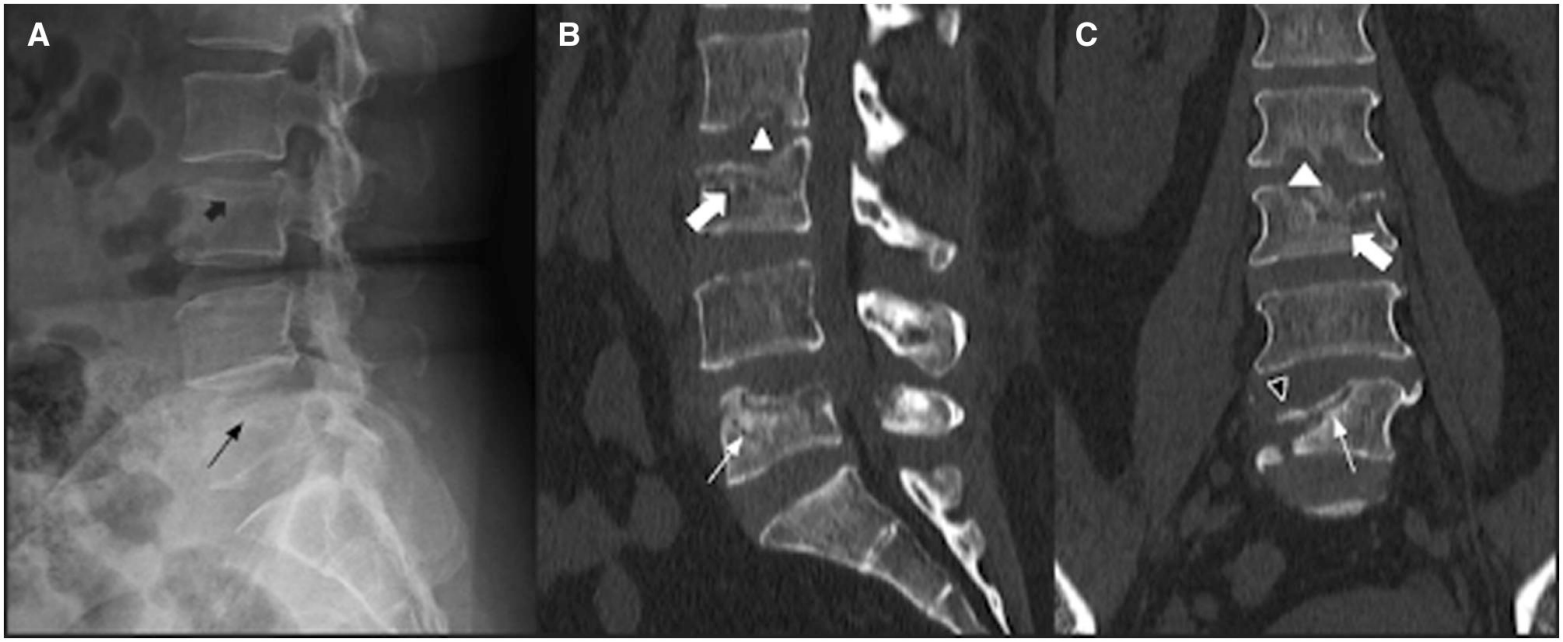
Plain radiograph and CT scan images of lumbosacral spine show subchondral endplate fractures with collapse of vertebral bodies—subchondral fracture of the superior endplate and reduced height of the L3 vertebra (black thick arrow) and irregular superior endplate with collapse of body and reduced height of the L5 vertebra (black thin arrow) is seen on a lateral radiograph (A), Subchondral fracture of inferior endplate of L2 (white arrowhead), subchondral fractures of superior endplate of L3 and L5 vertebra with reduction in height (white thick and thin arrows) and collapse of the right lateral aspect of the L5 vertebral body (black arrowhead) seen on sagittal and coronal plane images of CT scan (B) and (C).

*Imaging*: A plain radiograph of lumbosacral spine revealed subchondral fracture of the superior endplate of the L3 and L5 vertebrae with collapse of superior endplate, reducing the vertebral body height by 50% (Figure 3). As the patient was a known case of HIV and there was multilevel involvement the differentials for the radiograph findings included spondylodiscitis, osteonecrosis, osteoporotic fracture, and pathological fracture so an MRI scan was performed.

**Figure 4.**
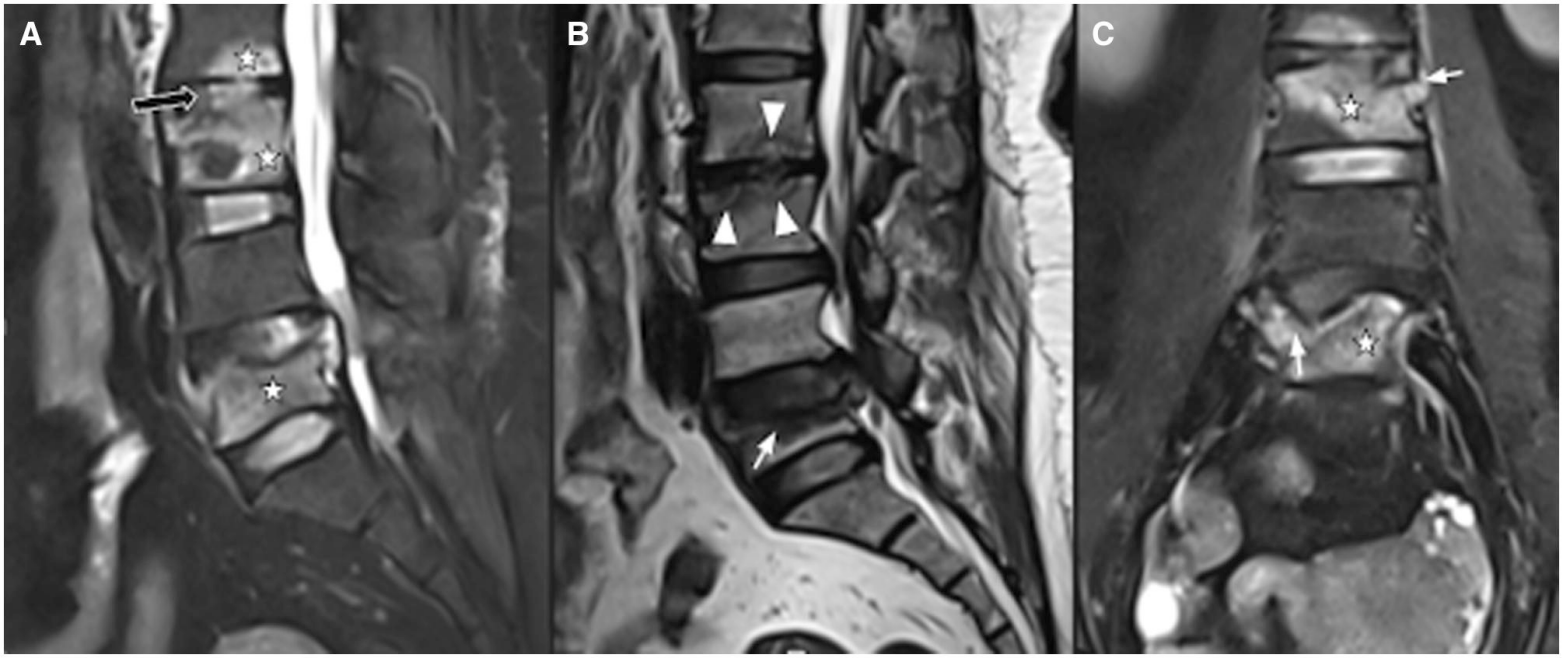
MRI of lumbar spine shows subchondral fractures in endplates of L2, L3, and L5 vertebra with collapse and marrow oedema, intradiscal fluid signal and double line sign**—**marrow oedema in the body of L2, L3, and L5 vertebrae (star), collapse with reduced height of L3 vertebra and near complete collapse of right lateral half of body of L5 vertebra (white arrow) and intradiscal fluid signal (black arrow) on sagittal and coronal STIR images (A) and (C). Subchondral fracture in L2, L3, and L5 vertebral body and double line sign in endplates of L2 and L3 vertebra (white arrowheads) on T2W image (B).

MRI images showed subchondral fracture of the inferior endplate of L2 and superior endplate of L3 and L5 vertebrae with reduced vertebral body height by >50%. Marrow oedema in the L2, L3, and L5 vertebral bodies. Fluid signal within the discs with diffuse disc bulge at L4-L5 causing severe spinal canal stenosis and compression over bilateral traversing L5 nerve roots. Minimal pre-and paravertebral soft tissue oedema. No features of pseudoarthrosis in the form of vacuum or fluid cleft. A double line sign was present in the adjacent endplate of L2 and L3 vertebra on T2W image. (Figure 4). On CT correlation, subchondral fractures of inferior endplate of L2, subchondral fracture of superior endplate of L3 and L5 vertebrae with a reduction in height and collapse of the right lateral aspect of the L5 vertebral body (Figure 3).

**Figure 5.**
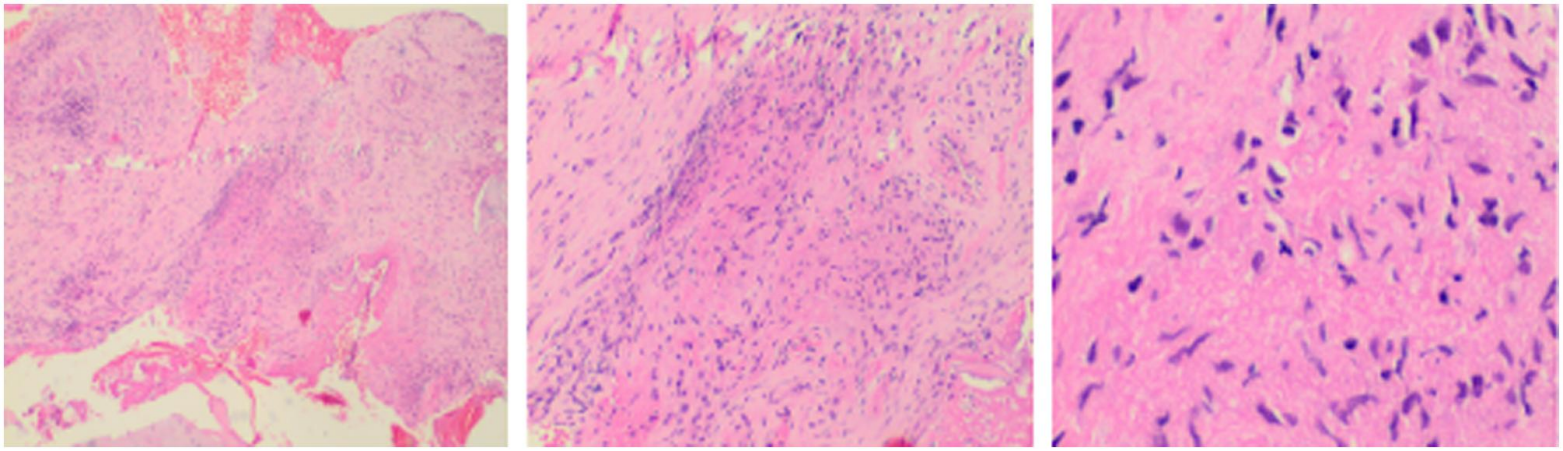
Histopathology slide images show acute on chronic inflammation with fibrinoid necrosis.

Due to significant soft tissue oedema and intradiscal fluid signal, a CT-guided biopsy was performed to rule out spondylodiscitis. Histopathological analysis following L5 vertebral body biopsy showed acute on chronic inflammation with fibrinoid necrosis without any evidence of infective or neoplastic features suggesting vascular pathology (Figure 5).

Analysing the history, clinical examination, laboratory investigations, imaging features, and histopathology report, subchondral fractures in multiple vertebral bodies with osteonecrosis were proposed as final diagnosis.

## Case 3

A 51-year-old gentleman presented to orthopaedic OPD with chief complaints of lower back pain for 4-5 months. The patient was a known case of HIV for 10 years. He was on medication for hypertension and type II DM for 8 years. The patient was a non-smoker and non-alcoholic with no other comorbidities like hepatitis B or C infection, or hyperparathyroidism.

Physical examination showed tenderness over the lumbosacral region on palpation and pain while all ranges of motion of the hip joint. A clinical suspicion of inflammatory aetiology was made.

*Lab investigation*: His absolute CD4 counts were 412 cells/mm^3^. The osteoporotic profile showed mild deficiency of vitamin D. The infective and inflammatory profile showed normal serum electrolytes, serum uric acid, RA factor, CRP, anti-Cyclic Citrullinated Peptide (CCP) antibody, and ESR.

**Figure 6.**
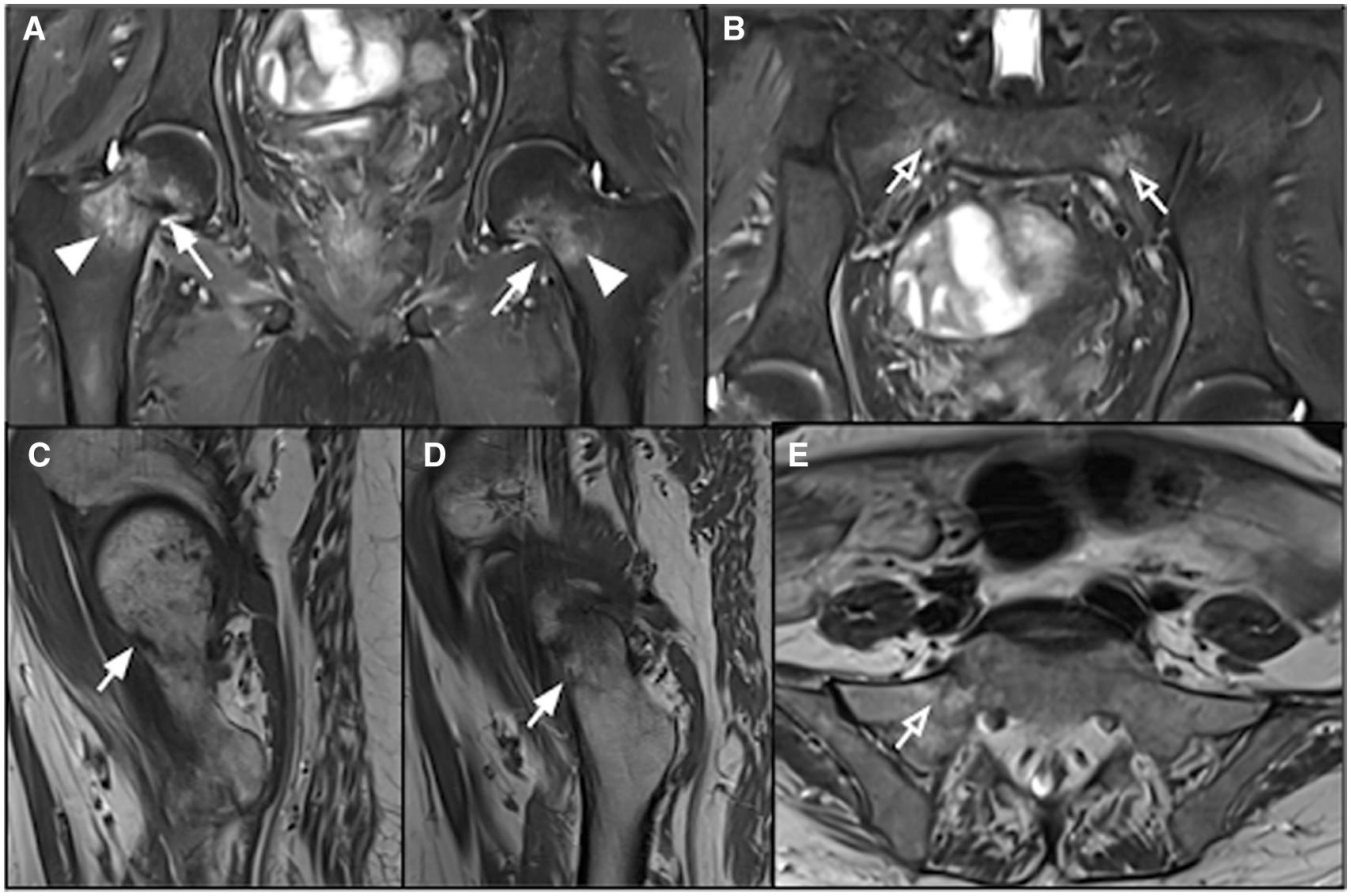
MRI pelvis showed insufficiency fractures in the femur neck and ala of sacrum on both sides—hypointense fracture line (white arrows) in neck of both femurs with surrounding marrow oedema (white arrowheads). Hypointense fracture line in both sacrum ala with surrounding marrow oedema (hollow white arrows) in coronal plane STIR image (A) and (B). Hypointense fracture line (white arrow) in neck of both femurs on sagittal plane T1W image (C) and (D). Hypointense fracture line in right sacral ala (hollow white arrow) with surrounding fat signal on axial planeT1W image (E).

**Figure 7.**
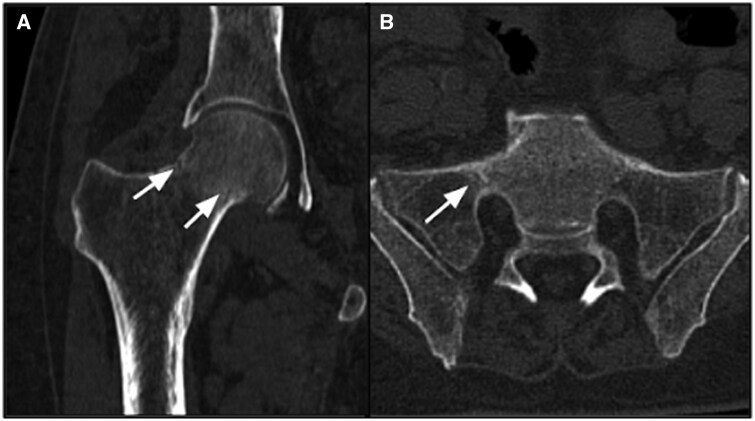
CT scan of pelvis shows insufficiency fractures in right femoral neck and ala of sacrum—fracture line in neck of right femur (white arrows) on coronal plane image (A). Thin fracture line is seen in the right ala of sacrum (white arrow) on axial plane image (B).

*Imaging*: MRI of pelvis and both hip joints showed hypointense fracture line in bilateral femur neck and both sacral ala with surrounding marrow oedema on STIR images (Figure 6). No marrow infiltrating lesion or intraosseous/soft tissue collection was appreciated. On CT scan, there was mild decrease in the bone density, a thin fracture line in the right sacral ala and fracture line in both neck of femur without any displacement (Figure 7).

On imaging, there was no evidence of any bone infiltrating lesion, soft tissue oedema, or collection and hence insufficiency fractures in bilateral femur neck and both ala of sacrum was proposed as final diagnosis.

On reviewing the drug regime of all 3 patients, they have been on tenofovir containing HAART for many years. The patients were advised immobilization, vitamin D3, and calcium supplementation and tenofovir plus emtricitabine was replaced by abacavir plus lamivudine. The pain was controlled gradually over a few weeks of follow-up.

## Discussion

The interaction of HIV infection and HAART affecting the bone health of the patient is a growing concern, as it affects the quality of life and morbidity of the patient. Our cases highlight the significant impact of tenofovir, a commonly used Anti retroviral therapy (ART) drug, on BMD and the associated increased risk of osteonecrosis and insufficiency fractures.

**Figure 8.**
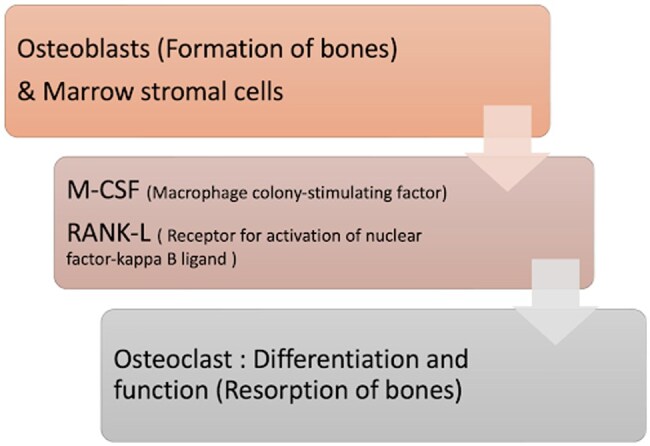
Normal regulation of bone remodelling.

The bone remodelling is regulated mainly by 2 cells in the body, osteoblast and osteoclast. Osteoblast functions for new bone formation and osteoclast functions for old bone resorption^2,^^3^ (Figure 8).

**Figure 9.**
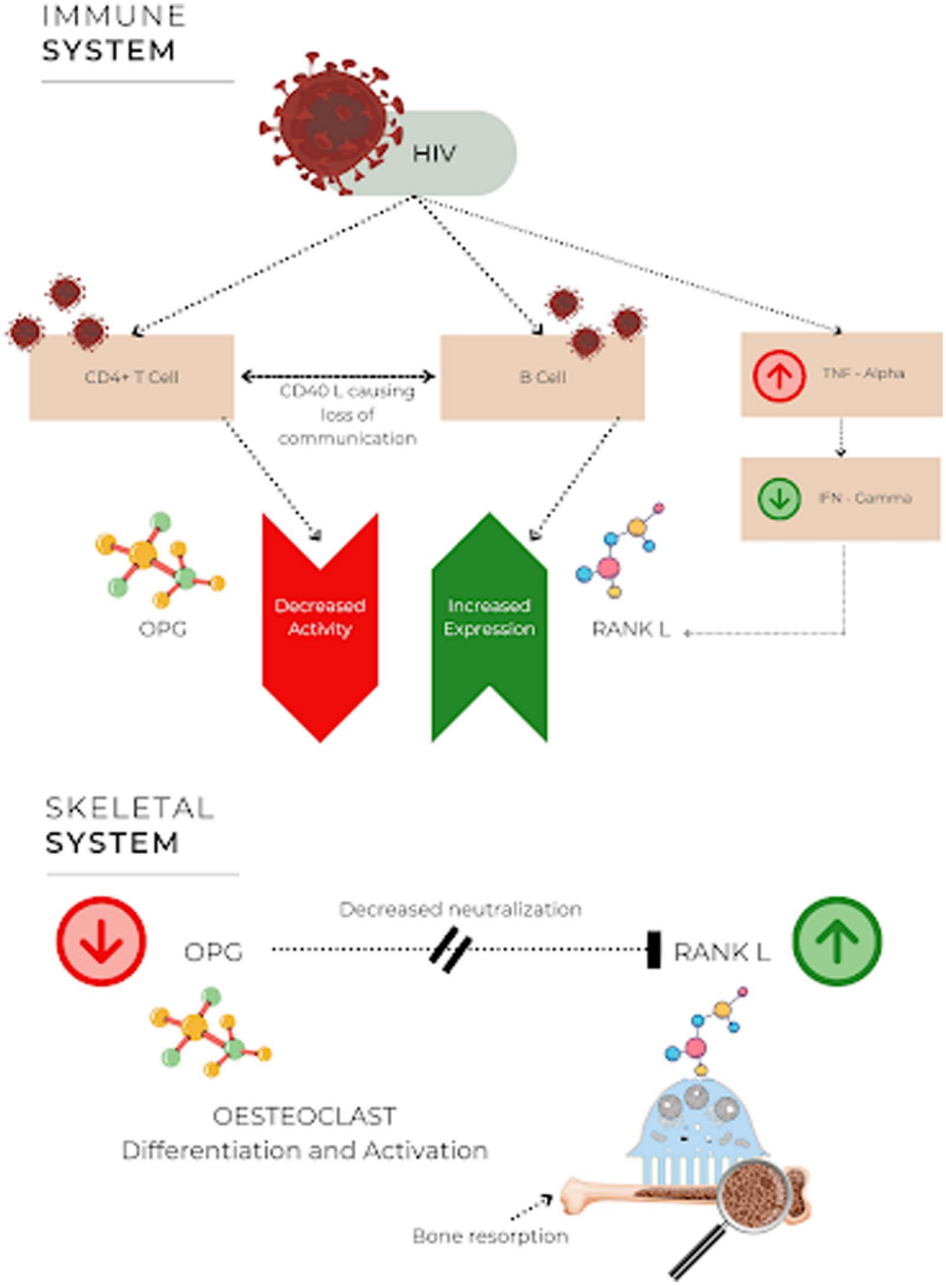
Effect of HIV on the immune system leading to activation of osteoclast.

Osteoclast function is influenced by multiple factors modulated during HIV-1 infection, which includes pro-inflammatory cytokines, such as TNF-α, RANK-L, osteoprotegerin (OPG), and IFN-gamma. This leads to loss of BMD. There is also disruption of T-cell to B-cell communication (Figure 9), which is achieved through CD40 ligand (CD40 L), leading to increased presence of RANK-L and diminished OPG production by B-cells. One of the important biomarker ratios is RANK-L/OPG. The increase in ratio is directly proportional to increase in bone resorption.^2-4^

**Figure 10.**
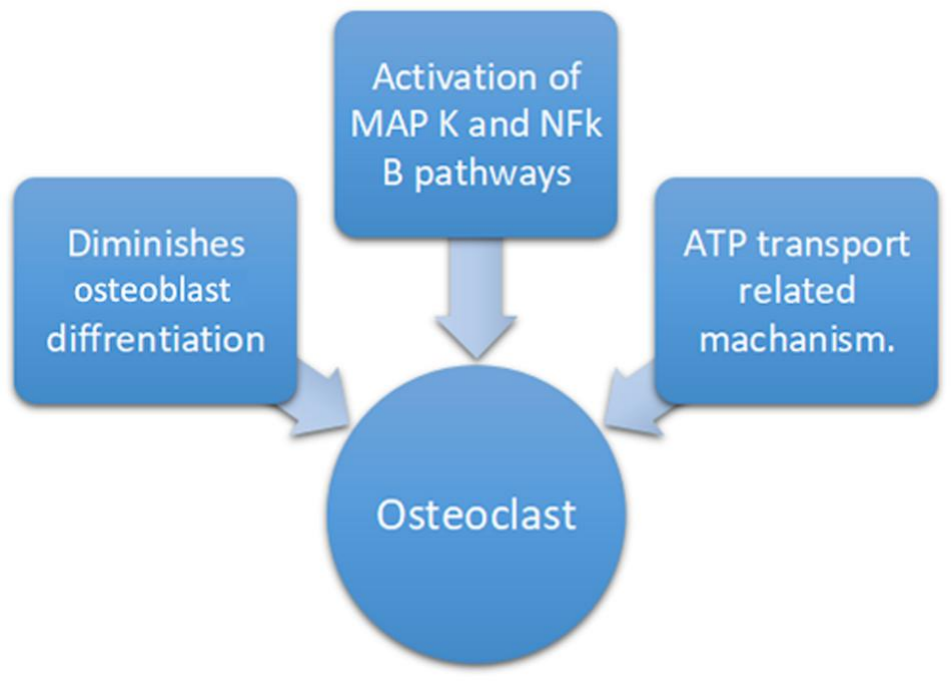
Various pathways through which tenofovir modulates osteoclast activation.

Studies have suggested that BMD changes due to Nucleside reverse transcriptase inhibitor (NRTIs), especially Tenofovir, abacavir, and zidovudine can be noted as early as 12 weeks in patients on NRTI-based regime.^5^ The precise mechanism of BMD reduction associated with tenofovir is unclear. Tenofovir directly promotes osteoclast differentiation and function through various pathways (Figure 10) resulting in increased expression of bone markers, such as cathepsin K, RANK-L, and NFATc1.^6^

Tenofovir is available in 2 variants: tenofovir disoproxil fumarate (TDF) and tenofovir alafenamide (TAF). TDF boosted with ritonavir or cobicistat is associated with higher risks of bone and renal (tubular necrosis) adverse events and lower HIV RNA suppression rates, compared with TAF.^7^

A meta-analysis suggested that exposure to tenofovir has a direct association with reduction in BMD and was an independent risk factor for developing fracture. However, no ART drug had been associated with the risk of developing osteonecrosis.^8^ Severe osteoporosis is known to eventually result in osteonecrosis and insufficiency fractures. However, no literature supporting the causal relationship between HAART and osteonecrosis was found.^9^

Both the European AIDS Clinical Society (EACS) and the Osteo Renal Exchange Program (OREP) recommend that fracture risk assessment should be performed in all HIV-infected individuals over 40 years of age using the FRAX algorithm. According to the EACS, those individuals with an estimated 10-year probability of major osteoporotic fractures ≥20% should undergo BMD measurement.^10^

Osteoporosis leads to insufficiency fractures, a type of stress fracture that occurs in a weak bone due to trivial force or trauma. Common sites of insufficiency fractures include sacrum, femoral neck, vertebrae, distal radius, pubic symphysis, tibia, and fibula.^11^ Common sites of insufficiency fracture in foot are second and third metatarsals, calcaneus, navicular, and talus respectively from most to least common.^12^ Subchondral fractures are stress fractures due to repeated microtrauma with intact overlying cartilage which differentiates them from osteochondral lesions.

Radiographs are the first imaging modality used for screening due to cost-effectiveness and easy availability. Various findings described in radiographs are cortex sign showing subtle loss of cortex density in early stages, with later stages having cortical thickening; solid periosteal reaction; and fracture line. However, radiographs have poor sensitivity for detecting stress fractures.^13^ A CT scan shows fracture line, periosteal reaction, sclerosis, and new bone formation, however in early stages these changes may not be appreciated well. MRI is the most sensitive modality for detecting stress fractures and differentiating it from cartilage and ligament injuries.^13^ Bone marrow oedema is the earliest stage followed by development of fracture line. Fluid sensitive sequences show linear hyperintense fluid cleft just below the fracture line and bone marrow oedema. T1W sequence shows hypointense fracture line. Another highly sensitive modality for detection of stress fractures is bone scintigraphy which shows hot spot at site of fracture.^14^ The dual energy CT scan detects bone marrow oedema increasing the overall sensitivity as compared to conventional CT scan.^15^

Several pathologies can present similar stress fractures both clinically and radiologically. In the infective process, there are oedematous changes in muscular plane and surrounding soft tissue, peripherally enhancing collections with diffusion restriction on MRI and intra-osseous collection. Solitary bone metastasis, a rare entity with incidence <2% will show marrow infiltrating lesion. The inflammatory process usually shows soft tissue oedema, synovial thickening, cartilage erosions, and lack of fracture line.

Most insufficiency fractures generally require conservative management like rest, ice application, elevation, brace application, and rehabilitation. When the fracture line has bi-cortical involvement, displaced fractured fragments, causes instability, or severe pain, surgical intervention is advised.

Osteonecrosis occurs due to vascular compromise predominantly involving epiphysis and metaphysis. It can be missed in initial stages on radiograph, later the hyperaemia of the surrounding bone causes focal osteopenia and necrotic fragment becomes sclerotic, may lead to subchondral crescent sign and articular collapse. On CT scan, in early stages, a clear fracture line can be seen with sclerosis of the fragment, later stages may show subchondral depression, collapse and fragmentation, degenerative changes in joint. MRI is most sensitive for early stages. Subchondral low signal on T1W images suggesting sclerosis. Fluid cleft and oedematous changes seen on fluid sensitive sequences. T2W images may display a typical double line sign which consists of a hypointense line depicting sclerosis and a hyperintense line depicting granulation tissue.^16^

Chronic bisphosphonate use can lead to osteonecrosis of the jaw, atypical subtrochanteric and femoral stress fractures, and generalized bone sclerosis resembling osteopetrosis. Atypical femoral fractures are usually transverse, involve the lateral cortex, occur with trivial or no trauma, may show a medial spike, lack comminution, and have localized endosteal or periosteal thickening. The osteoporotic fractures typically involve the femoral neck or intertrochanteric region. Jaw osteonecrosis shows mixed lucency and sclerosis, periosteal reaction, and adjacent bone destruction on radiographs and CT.^17^^,^^18^

Vertebral osteonecrosis is also known as Kummel’s disease. The imaging features include collapse of vertebrae, fluid cleft, vacuum phenomena (not present in all patients), and bone marrow oedema on MRI. Late subacute to chronic cases may show sclerosis. Presence of vacuum is associated with higher amount of instability and higher amount of collapse.^19^

There is several-fold increased risk of developing osteonecrosis in peripheral bones in a patient with HIV infection. Vertebra is a rare site for osteonecrosis. More common in females and adults with age more than 50 years. It usually involves a single vertebra from T8 to L4 vertebral level. Only few case reports have been published till date with patients having multiple vertebral body involvement.^20^

The main differential diagnosis of VON is the osteoporotic vertebral fracture. It is important to differentiate these conditions because VON is more frequently associated with the development of gross kyphotic deformity and neurological complications because of posterior wall displacement. However, this differentiation may be challenging because of difficulty in detecting due to overlapping imaging features and both conditions may even coincide in the same patient.^19^ Fluid sign is specific for osteoporotic compression fractures. Pathological fracture features include multiple lesions, lytic/sclerotic lesion, marrow infiltration, epidural/paraspinal mass. Formica et al devised a classification system for treatment planning for VON. Stage 0 (theoretical phase), stage 1 (early phase), stage 2 (instability phase), and stage 3 (fixed deformity phase) which can be easily identified on imaging.^21^

We wanted to draw attention towards the deleterious effect of the HIV infection and antiretroviral therapy (tenofovir) on BMD and its consequences. Also emphasizes the need for monitoring and proactive management of bone health in patients on long-term ART. As our understanding of the effects of HIV and its treatment on the musculoskeletal system evolves, clinicians must remain vigilant in assessing fracture risk and implementing preventative strategies.

## Conclusion

Patients with HIV on HAART, particularly those using tenofovir-containing regimens, experience a significant reduction in BMD. This correlates directly with several factors: the duration of HIV infection, the length of time on antiretroviral therapy, and the specific class and molecular variant of the antiretroviral drugs used.

The cumulative effects of HIV and HAART can lead to severe osteoporosis, which increases the risk of insufficiency fractures and osteonecrosis. Notably, severe osteoporosis can also act as a precursor to osteonecrosis. Imaging plays a major role in the detection of these changes and treatment monitoring.

## Informed consent

A written informed consent was obtained from the patients for publication of this case review, including accompanying images.

## Learning points

- Every HIV patient presenting to the outpatient department should not be considered as a infection case.
- In HIV patients presenting with osteopenia, insufficiency fracture or osteonecrosis, the drug regime should be carefully checked for tenofovir and required modifications should be done.
- Prolonged tenofovir containing regime accelerates bone loss making the bone prone to insufficiency fractures and osteonecrosis. So patients on prolonged tenofovir containing regime should undergo regular bone mineral density assessment and managed accordingly.
